# Regulation of E2F1 activity via PKA-mediated phosphorylations

**DOI:** 10.3906/biy-2003-9

**Published:** 2020-10-13

**Authors:** Mustafa Gökhan ERTOSUN, Sayra DİLMAÇ, Fatma Zehra HAPİL, Gamze TANRIÖVER, Sadi KÖKSOY, Osman Nidai ÖZEŞ

**Affiliations:** 1 Department of Plastic, Reconstructive, and Aesthetic Surgery, Faculty of Medicine, Akdeniz University, Antalya Turkey; 2 Department of Histology and Embriology, Faculty of Medicine, Akdeniz University, Antalya Turkey; 3 Department of Medical Biology and Genetics, Faculty of Medicine, Akdeniz University, Antalya Turkey; 4 Department of Medical Microbiology, Faculty of Medicine, Akdeniz University, Antalya Turkey; 5 Altay Therapeutics, San Bruno, CA USA

**Keywords:** E2F1, cell cycle, PKA, forskolin, senescence, proliferation

## Abstract

E2F1 becomes activated during the G1 phase of the cell cycle, and posttranslational modifications modulate its activity. Activation of G-protein coupled receptors (GPCR) by many ligands induces the activation of adenylate cyclases and the production of cAMP, which activates the PKA enzyme. Activated PKA elicits its biological effect by phosphorylating the target proteins containing serine or threonine amino acids in the RxxS/T motif. Since PKA activation negatively regulates cell proliferation, we thought that activated PKA would negatively affect the activity of E2F1. In line with this, when we analyzed the amino acid sequence of E2F1, we found 3 hypothetical consensus PKA phosphorylation sites located at 127-130, 232-235, and 361-364 positions and RYET, RLLS, and RMGS sequences. After showing the binding and phosphorylation of E2F1 by PKA, we converted the codons of Threonine-130, Serine-235, and Serine-364 to Alanine and Glutamic acid codons on the eukaryotic E2F1 expression vector we had previously created. We confirmed the phosphorylation of T130, S235, and S364 by developing monoclonal antibodies against phospho-specific forms of these sites and showed that their phosphorylation is cell cycle-dependent. According to our results, PKA-mediated phosphorylation of E2F1 by PKA inhibits proliferation and glucose uptake and induces caspase-3 activation and senescence.

## 1. Introduction

E2F family members play an important role in the regulation of the cell cycle and apoptosis. The first E2F family member, later named E2F1, was identified as a protein involved in the transcriptional regulation of the adenoviral E2 gene (Kovesdi et al., 1986; Hazar-Rethinam et al., 2011). After cloning the rest of the 7 members of the E2F family and showing their associations with pRB, the E2F family member was placed to the center of the regulation of the cell cycle and development of cancer (Bandara and La Thangue, 1991; Kaelin et al., 1992). E2F family members are classified as transcriptional activators (E2F1, E2F2, and E2F3a), repressor (E2F3b, E2F4, and E2F5), and inhibitor (E2F6, E2F7a, E2F7b, and E2F8). The expression of activator E2F members is regulated by the cell cycle, and their level reaches to a maximum at the G1/S phase of the cell cycle (DeGregori et al., 1995). Although activator E2Fs are transcriptionally induced during the G1/S phase, the repressor E2Fs are kept at basal levels through the cell cycle and suppress the expression of genes required during the S phase (Takahashi et al., 2000). Meanwhile, the inhibitor E2Fs competitively bind to both the activator and the inhibitor E2Fs and modulate the cell cycle differently, depending on the cellular context (Iaquinta and Lees, 2007).

E2F1 binds to the TTTSSCGCS consensus sequence at the promoter of target genes by dimerizing with its dimerization partners (DP) (Helin et al., 1993). The target genes induced by E2F1 include CDC2, CDC25A, cyclin D1, cyclin E, DHFR, DNA polymerase alpha, replication factor-alpha (RFC3), and RAD52 (Slansky et al., 1993; DeGregori et al., 1995; Ohtani et al., 1995; Inoshita et al., 1999; Muller et al., 2001; Stanelle et al., 2002). In addition to its role in the G1/S phase, E2F1 also plays an essential role in slowing down the cycle in the absence of nutrients and growth factors (Shan and Lee, 1994). Besides, E2F1 induces the p53-dependent and independent apoptosis by inducing the expression of p53, ARF, Apaf1, ASPP1/2 (apoptosis stimulating protein of p53), and p73 (Qin et al., 1994; Shan and Lee, 1994; Wu and Levine, 1994; Irwinet et al., 2000; Lindstrom and Wiman, 2003; Chenet et al., 2005) and by suppressing the expression of NF-kB (Ma et al., 2002).

In addition to its role in the regulation of the cell cycle and apoptosis, E2F1 also controls the proliferation/differentiation of neuronal cells and the development of the neocortex (Mohan et al., 2012). Furthermore, E2F1 controls lipid metabolism by inducing the transcription of SREBP (sterol regulatory element-binding protein) and PPARg (Dali-Youcef et al., 2007). Also, E2F1 controls the synthesis and secretion of insulin from the pancreatic beta cells by inducing the expression of Kir6.2 (Blanch et al., 2011), while also repressing glucose metabolism by inducing the expression of pyruvate dehydrogenase kinase 4 (PDK4) (Hsieh et al., 2008). E2F1 inhibits VEGF expression under hypoxic conditions by inducing p53 (Berger et al., 2010), and induces expression of VEGF receptors FLT1 (VEGFR1) and KDR (VEGFR2) under oxygenic conditions (Pillai et al., 2010) and directly regulates the expression of MMP9, MMP14, and MMP15 (Johnson et al., 2012).

Until 1989, it was largely accepted that the activity of E2F1 was regulated by its inhibitor, pRB. In 1989, Bagchi et al. showed that E2F1 can be activated by phosphorylation (Bagchi et al., 1989). However, in the following years, several groups of researchers showed that CDK-mediated phosphorylation of E2F1 at different residues can either increase or decrease its DNA and pRB binding affinities (Peeper et al., 1995; Zhao et al., 2013). It was later shown that during DNA damage, ATM and CHK2-mediated phosphorylations of E2F1 at Serine 31, 364 facilitate the binding of E2F1 to the promoters of proapoptotic genes as well as 14.3.3 (Lin et al., 2001; Stevens et al., 2003). The phosphorylation of E2F1 at Serine 403 and Threonine 433 has been shown to be carried out by different enzymes such as p38, JNK1, and glycogen synthase kinase-3β (GSK3β). The phosphorylation of these induces nuclear export and stabilization of E2F1 (Wang et al., 1999; Garcia-Alvarez et al., 2007; Ivanova et al., 2009).

In addition to phosphorylation, E2F1 also undergoes acetylation at Lysine 117, 120, and 125 by p300, P/CAF, and Tip60; these acetylations increase the ability of E2F1 to bind to ADA2 and ASC2 (Marzio et al., 2000). E2F1 uses the negative-feedback mechanism to control its own acetylation by inducing the expression of deacetylase SIRT1. When SIRT1 activity is inhibited, increased cell death is observed in the p53-deficient cells after DNA damage (Pediconi et al., 2009; Olmos et al., 2011). Although phosphorylation and acetylation positively stimulate the function of E2F1, E2F1 can also be methylated at Lysine 185, Arginine 111/113. All methylations induce ubiquitination and the degradation of E2F1, while demethylated by lysine-specific demethylase (LSD1) causes the stabilization of E2F1 (Kontaki and Talianidis, 2010). Recently, it was shown that E2F1 undergoes neddilation and ADP-ribosylation. Neddylation causes destabilization of E2F1 and prevents the proapoptotic and transcriptional activity of E2F1 during DNA damage. NEDD8 is removed by Sentrin-cysteine protease 8 (SENP8), and this reactivates E2F1 to induce the transcription of proapoptotic genes (Aoki et al., 2013). PARP1-mediated poly-ADP-ribosylation of E2F1 results in a pause in the cell cycle or induction of apoptosis in the presence or absence of growth factors, respectively (Kumari et al., 2015). 

Cyclic AMP-activated kinase (PKA) is known for its antiproliferative properties and claimed to be mediated by different means. For example, Schmitt et al. showed that cAMP-mediated inhibition of cell growth is mediated by the phosphorylation of SRC by PKA (Schmitt and Stork, 2002). Ferretti et al. showed that PKA is activated shortly after the removal of glucose and activated PKA phosphorylates AMPK and induces its antiproliferative activity (Ferretti et al., 2016). Similar to this, it was recently shown that GPCR-activated PKA phosphorylates the raptor subunit of mammalian mTORC1 and modulates the growth-promoting activity of mTORC1 (Jewell et al., 2019). In addition to all of these findings, several publications have also showed that the antiproliferative activities of adiponectin, forskolin, and sildenafil are mediated by the activation of PKA by these molecules (Tantini et al., 2005; Medina et al., 2014).

As mentioned above, E2F1 can perform a dual function during the cell cycle and can promote cell growth or apoptosis, depending on the conditions of growth media. Most of these dual functions seem to be modulated by posttranslational modifications. Given that the activation of PKA can have significant impacts on cell proliferation and that proliferation is tightly regulated by the cell cycle, we hypothesized that E2F1 could be a target of PKA as E2F1 is primary responsible for the progression of the cell cycle from the G1 to S phase. Indeed, when we analyzed the amino acid sequence of E2F1, we found 3 putative PKA phosphorylation motifs (RXXS/T) located at T130, S235, and S364. Therefore, we first tested whether the PKA activator (forskolin) would affect the cellular level of E2F1 and found that PKA activation with 20-uM forskolin gradually reduced the cellular level of E2F1 for at least 16 h. After this promising data, we mutated the codons of the hypothetical PKA phosphorylation sites T130, S235, and S364 to nonphosphorylatable Alanine and to phosphorylate the mimicking Glutamic acid. We then tested the effect of these mutants on cell proliferation, expression of Ki67, induction of apoptosis, and glucose uptake and senescence. Collectively, our results indicate that while Glutamic acid mutants suppress proliferation, Alanine mutants induce it, indicating that PKA-mediated phosphorylation of E2F1 shifts its activity. In line with this, while Glutamic acid mutants induce the induction of apoptosis, Alanine mutants repress apoptosis.

## 2. Materials and methods

### 2.1. Cell culture and biological reagents

Reagents were obtained from the following sources: monoclonal anti-E2F1 and anti-GAPDH came from Santa Cruz Biotechnology, Inc. (Santa Cruz, CA, USA). The monoclonal anti-GAPDH antibody was also from Santa Cruz Biotechnology, Inc.; antirabbit HRP and antimouse HRP were purchased from Bio-Rad Laboratories, Inc. (Hercules, CA, USA). HEK293 cells were grown in DMEM supplemented with 10% FBS, 100 mg/mL penicillin, 50 µg/mL streptomycin, and 1 mM glutamine.

### 2.2. Cloning and side directed mutagenesis

Cloning primers:

Forward: 5’-ccggaattcgccgccatggccttggccggggcccctgcggg-3’

Reverse: 5’-ccggaattcgaaatccaggggggtgaggtccccaaagtc -3’ 

Mutagenesis primers:

T130,S235, and S364 of human E2F1 were mutated to Alanin (T130A,S235A, and S364A) and Glutamic Acid (T130E,S235E,S364E) with the Pfu polymerase (Thermo Fisher Scientific Inc.,Waltham, MA, USA) using the primers listed above. PCR conditions were 30 s at 95 °C, followed by 18 cycles of 95 °C for 30 s, 56.5 °C for 1 min, 72 °C for 10 min, and 1 step of 72 °C for 10 min. Mutations were verified by DNA sequencing (Supplemental Figures S1–S3).

T130A Forward: 5’-agaagtcacgctatgaggcctcactgaatctgacc-3’ 

T130A Reverse: 5’-ggtcagattcagtgaggcctcatagcgtgacttct-3’

S235A Forward: 5’-ctgcgcctgctcgccgaggacactg-3’

S235A Reverse: 5’-cagtgtcctcggcgagcaggcgcag-3’

S364A Forward: 5’-tcccggatgggcgccctgcgggctcc-3’

S364A Reverse: 5’-ggagcccgcagggcgcccatccggga-3’

T130E Forward: 5’-gggagaagtcacgctatgaggagtcactgaatctgaccaccaa-3’

T130E Reverse: 5’-ttggtggtcagattcagtgactcctcatagcgtgacttctccc-3’

S235E Forward: 5’-cagctgcgcctgctcgaggaggacactgacagc-3’

S235E Reverse: 5’-gctgtcagtgtcctcctcgagcaggcgcagctg-3’

S364E Forward: 5’-gtcccggatgggcgagctgcgggctcccg-3’

S364E Reverse: 5’-cgggagcccgcagctcgcccatccgggac-3’

### 2.3. Transfections and treatments

Sixty to seventy percent confluent HEK293 cells were transfected with 15 µg of mock or E2F1 expression vectors prepared in pcDNA3.1. After 16 h, the cells were shocked with medium containing 10% glycerol and cultured in complete DMEM for 24 h. The cells were then serum-starved for 16 h and treated with reagents.

### 2.4. Immunoprecipitation and Western blot

Protein concentrations of whole cell lysates obtained in lysis buffer [20 mM Tris-HCl (pH 7.4), 150 mM NaCl, 1.2% Triton X-100, 1 mM EGTA, 1 mM EDTA, 1 mM PMSF, 0.15 U/mL aprotinin, 10g/mL leupeptin, 10g/mL pepstatin A, and 1 mM sodium orthovanadate] were determined using the Bradford method. Before immunoprecipitations, lysates were precleared with 50-μL protein A/G-Agarose beads (Santa Cruz Biotechnology, Inc., sc-2003). To precipitate the protein complexes, 2-μg primary antibody was added per 1-mg protein lysate, incubated for 2 h in +4 °C nutator; then, protein A/G-agarose beads were added and incubated overnight in +4 °C nutator. Beads were pelleted by centrifugation at 6000×g for 1 min, washed in cold lysis buffer at least 3 times, resuspended in SDS sample buffer containing β-mercaptoethanol and boiled for 5 min. Equal amounts of protein were fractionated by SDS-PAGE on 10% polyacrylamide gels and transferred overnight to Immobilon-P PVDF membranes. Blots were probed with primary antibodies and corresponding HRP-conjugated secondary antibodies, and proteins were detected using Clarity ECL western blotting substrate (Bio-Rad Laboratories, Inc., 1705061). 

### 2.5.Cell viability testing and MTT procedure

To investigate the effects of wild type and mutants of E2F1 on proliferation of HEK293 cells, 3000 HEK293 cells were reverse transfected with 300-ng vectors with lipofectamine 2000 in 96-well plates with 6 replicates in complete DMEM. The cells were then incubated for 72 h. At the end of the 72 h, 20 µL of MTT (5 mg/mL) solution was added for 4 h at 37 °C in an incubator. The medium was then removed, and DMSO (100 µL) was added to dissolve the formazan crystals. The plates were shielded from light using a foil and left in an orbital shaker maintained at 600 revolutions/minute for 5 m. The amount of MTT formazan product formed was determined by measuring absorbance at 540 nm, with 690 nm as the reference wavelength.

### 2.6. Immunofluorescent in cell cultures: Ki67 staining

Ki67 (proliferation marker) staining in transfected HEK293 cells was done by immunofluorescence (IF) assay. Briefly, HEK293 cells were transfected with vectors and 24 h after transfection, the cells were plated at a density of 3000 cells per well in a 96-well microtiter plate with 6 replicates. The cells were incubated for 72 h. After this, fixed cells were incubated with primary antibody overnight at 4 °C, followed by 1-h incubation with Alexa Fluor 594 (Jackson ImmunoResearch Laboratories, Inc., West Grove, PA, USA) in the dark at 37 °C. Ki-67 monoclonal antibody (cell signalling) was used at dilutions of 1:200 and 1:800, respectively. Species- and subtype-matched antibodies were used as negative controls [universal negative controls for mouse or rabbit primary antibodies (Dako Denmark A/S, Glostrup, Denmark)]. The coverslips were mounted on the slides by using a Slow Fade Gold antifade reagent with 4′,6-diamidino-2-phenylindole (DAPI) (Life Technologies Co., Carlsbad, CA, USA). Images from the IF slides were obtained with an inverted microscope (Olympus IX51) with 20× magnification. For quantifying the number of Ki67+, 3 pictures were randomly taken from each sample in a blind manner. 

### 2.7. Caspase 3 activation assays

Caspase 3 activity was assessed with colorimetric caspase assay kits (Bio vision K106-200), according to the manufacturer’s instructions. Briefly, HEK293 cells were transiently transfected with vectors; at the 48th hour of transfection, the cells was lysed with cellular lysis buffer, then protein concentrations were determined. Approximately 150-μg lysate volume was brought to 50 uL with cell lysis buffer, and 50-uL 2X reaction buffer containing 10 mM of DTT was added. Lysates were either incubated DEVD-pNA (200 uM) or IETD-pNA (200 uM) for 2 h, and colorimetric measurements were taken at 405-nm wavelength. 

### 2.8. Glucose uptake assay

To determine whether Alanine and Glutamic acid mutants would affect the glucose uptake after insulin stimulation, we used a cell-based Glucose Uptake Assay Kit (Cayman Chemical Company, Ann Arbor, MI, USA). Briefly, 3000 HEK293 cells were reverse transfected with 300-ng vectors with lipofectamine 2000 in 96-well plates with 3 replicates in complete DMEM and cells were incubated for 24 h; at the end of incubation, we washed the cells with glucose-free medium (Sigma-Aldrich Corp., St. Louis, MO, USA, D5030). Then, the cells were incubated with glucose free medium containing glucose analog 2-NGBD in the presence and absence of insulin (100 ng/mL) in the dark; subsequently, the medium was removed, the cells were washed, and the 2-NBDG taken up by the cells was detected using fluorescent filters with excitation 485 nm and emission 535 nm. Fluorescein intensity was calculated by using ImageJ analysis program. 

### 2.9. Senescence assays

SA-β-Gal staining was performed using an Abcam Kit ab65351 (Abcam PLC, Cambridge, UK) according to the manufacturer’s specifications. Images were collected using a Zeiss Primo Star microscope (Carl Zeiss Microscopy GmbH, Oberkochen, Germany). The fraction of SA-β-Gal–positive cells was determined by counting the total and SA-β-Gal–stained cell’s area in 3 to 6 fields (×40) per treatment per cell line via an ImageJ analysis program.

### 2.10. Thymidine block (cell cycle arrest) and cell cycle analysis

HEK293 cells were synchronized into the G1 phase by releasing mitotic cells for 8 h, into S phase by a double thymidine block (after incubation with 2 mM thymidine for 16 h, the cells were washed and released into complete media for 8 h, followed by another 16h of incubation with 2 mM thymidine), and into the G2 by releasing S phase cells for 8 h. After thymidine blocking, the cells were treated with 10% FBS including medium for the specified time. Cell cycle analysis was monitored by flow cytometry by FACSCanto II (BD). Cells were fixed in 70% ethanol and treated with RNase (100 µg/mL) before staining with propidium iodide (50 μg/mL; Sigma-Aldrich Corp.). The cells were then gated and the histogram function was plotted in /(BD).

### 2.11. Development of monoclonal antiphospho E2F1 antibodies

Antibodies were made as previously described (Özeş et al., 2018). Phosphopeptides of E2F1 (5-GVKSPGEKSREY-pT130-SLNLTTKR-3,5’ HLMNICTTQLRLL-pS235-EDTDSQR-3’: 5’-LEQEPLLSRMG-pS364-LRAPVDEDR-3) were manufactured by IT-Technologies. Approximately 100 ng of peptides dissolved in sterile water was mixed with 100 ng of LPS, 100 uL of mineral oil, and then vortexed for 30 s. Balb/C mice were immunized intraperitoneally twice a week for 8 weeks. At the end of the 8 weeks, the animals were sacrificed and their spleens were removed; hybridomas were created with Fo cells using PEG-4000. Then, 1/1000 times diluted hybridomas were transferred in 96-well plates in 200 µL. The next day, wells containing a single cell were marked, and these cells were incubated for 2 weeks. At the end of this period, the cells were transferred in T25 and further incubated for 30 days. Supernatants of these cells were collected and antibodies in supernatants were purified by using Amicon Pro Affinity Concentration Kit Protein A with 100 kDa colon (ACK5100PA).

### 2.12. Statistical analysis

Statistical software SPSS and Graphpad Prism were used. Comparison of parameters was performed using the Wilcoxon signed-ranks test. A P value of <0.05 was considered to be statistically significant.

## 3. Results

### 3.1. PKA activation destabilizes E2F1 and mutants of E2F1 can be ectopically expressed

**Figure 1:**
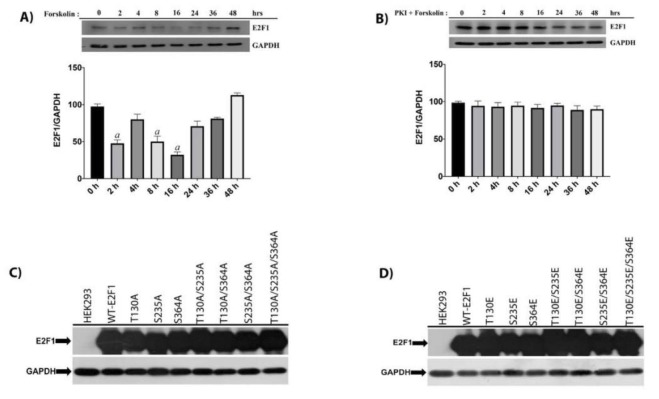
PKA activation destabilizes E2F1 and mutants of E2F1 can be ectopically expressed. (A) HEK293 cells were treated with forskolin (20 mM) and cellular lysates were collected for indicated times. Approximately 100-mg total protein lysates were fractionated on 10% SDS-PAGE and blot was labeled with an anti-E2F1 antibody. The blot was stripped off and relabeled with an anti-GAPDH antibody. Band intensities were determined using ImageJ, and the relative abundance of the E2F1 level was determined by dividing E2F1 band intensities to that of GAPDH. (B) HEK293 cells were pretreated with PKA inhibitor H9 for 1 h, then forskolin was added for indicated times and levels of E2F1; GAPDH relative change in expression of E2F1 was determined as mentioned. Band intensities were determined using ImageJ and relative abundance of E2F1 level was determined by dividing E2F1 band intensities to that of GAPDH. (C,D) Subconfluent HEK293 cells cultured DMEM in 100 mm plates were transfected with 30-ug expression vectors of Alanine mutants (C) and Glutamic acid mutants (D) of E2F1 using calcium phosphate protocol for 48 h. Cellular lysates were collected in lysis buffer and 100-ug total protein lysates were fractionated on 10% SDS-PAGE, and the blot was labeled with an anti-E2F1 antibody. The blot was stripped off and relabeled with an anti-GAPDH antibody (WT-E2F1 was used as a control in statistical analysis. “a”: statistically significant decrease, P < 0.0001).

Since we postulated that E2F1 could be phosphorylated at 3 different sites by PKA, we wanted to determine whether this phosphorylation would have any effect on the expression/stability of E2F1. For this, we first stimulated HEK293 cells, grown in regular DMEM containing 10% serum, with 20 mM of forskolin and prepared total cellular lysates and determined the level of E2F1 by western blotting. As shown in Figure 1A, forskolin treatment caused a sharp drop in the E2F1 level during the 2nd hour of poststimulation. Compared to the control cells, forskolin treatment caused a slight increase at the 4th hour and a gradual decrease of nearly 75% during the 16th hour of poststimulation. After the level during the 16th hour, E2F1 slowly increased and reached to control levels at the 48th hour of poststimulation. To validate whether the observed results were due to PKA activation, we preincubated HEK293 cells with a well-known PKA inhibitor H9 (2.5mM) for 1 h before stimulation with forskolin. As shown at Figure 1B, preincubation of HEK293 cells with H9 completely abrogated the forskolin-induced E2F1 downregulation/degradation. These results clearly indicate that forskolin stimulation and activation of PKA either destabilizes E2F1 or represses its expression. Since we generated Alanine and Glutamic acid mutants of E2F1, we wanted to show whether or not introduced mutations would affect the ectopic expression/stability of our mutants. To show this, we reverse transfected HEK293 cells with 6 µg of vectors using Lipofectamine 2000 for 48 h, prepared total cell lysates, and determined the level of E2F1 by western blotting. As shown in Figures 1C and 1D, both Alanine and Glutamic Acid mutants can be expressed at very high levels for at least 48 h posttransfection. The blots were overexposed in order to determine the level of fold induction of mutants compared to the level of endogenous E2F1. However, due to very high levels of expression of mutants, we could not detect endogenous E2F1 at the time the bands of the mutants were already bleached out. Since we determined that expression of endogenous E2F1 is very low compared to mutants, we did not try to suppress the expression of endogenous E2F1 by si- or shRNA targeting E2F1 for the remaining experiments.

### 3.2. PKA and E2F1 binds to each other and activation of PKA causes decline in the protein level of E2F1 

**Figure 2:**
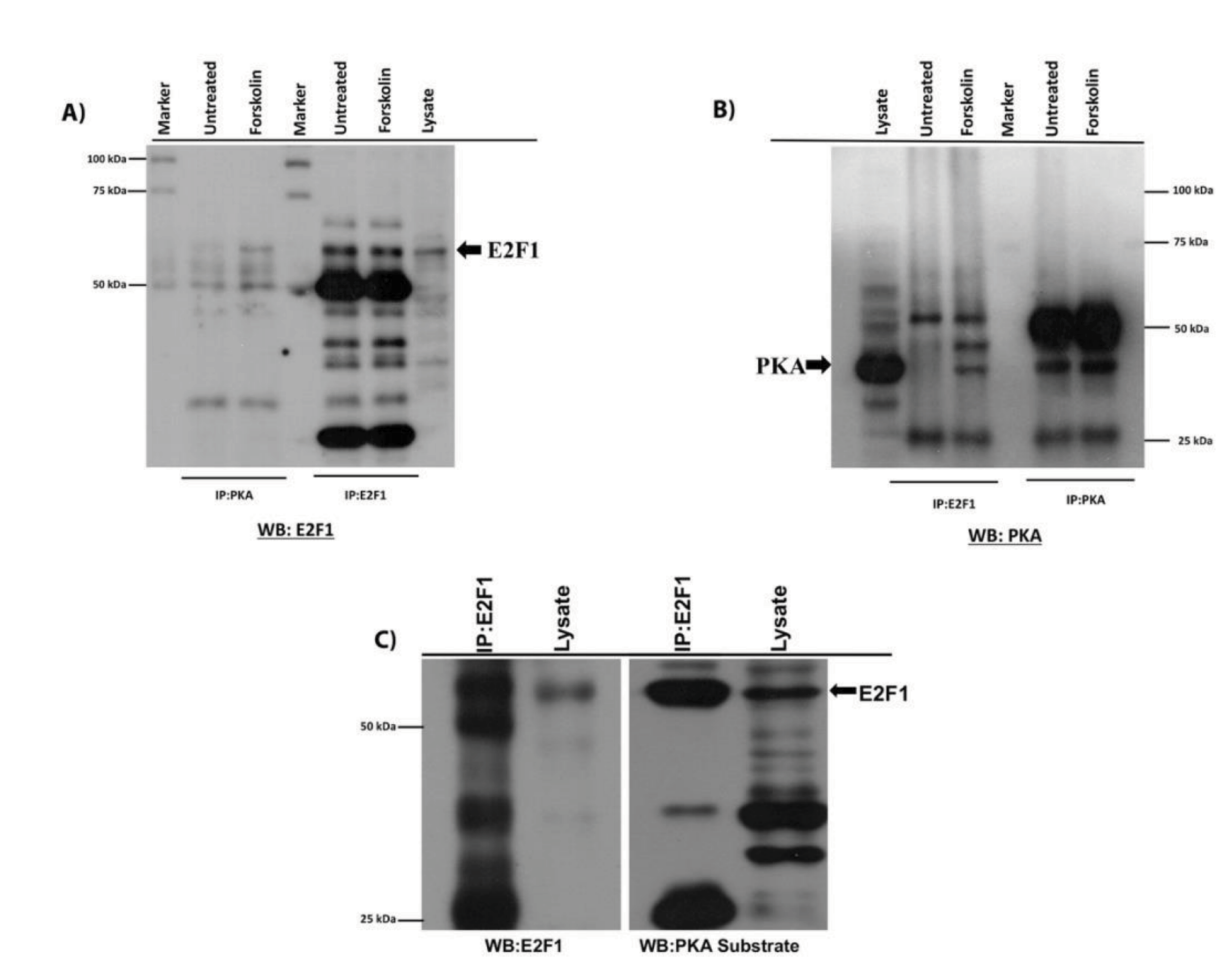
PKA and E2F1 bind to each other, and activation of PKA causes a decline inthe protein level of E2F1. (A,B) Physical interaction between E2F1 and PKA was explored with reciprocal immunoprecipitations. E2F1 and PKA were immunoprecipitated from untreated and 1 h- forskolin-treated HEK293 cells. E2F1 and PKA were immunoprecipitated from 1mg of total cellular lysate and blotted with E2F1 and PKA antibodies. (C) In vitro kinase reaction was performed with a PKA catalytic subunit. Wild type E2F1 was immunoprecipitated from 3-mg total cellular lysate prepared from HEK293 cells overexpressing the wild type E2F1. After immunoprecipitation, the pellet was washed 3 times in 1X PKA reaction buffer and resuspended in 50 mL of reaction buffer. It then underwent in vitro kinase reaction with the addition of the PKA catalytic subunit PKAca (purchased from NEB-P600S) and 10 mM ATP. The reaction mix was incubated for 30 min at 37oC. We terminated the reaction by incubating the samples at 65 °C for 5 min, then washed the reaction mix according to the manufacturer’s protocol; we then resuspended the pellet in SDS-loading dye, incubated it at 95 °C for 5 min, and fractionated the supernatant and lysate in duplicates using 10% SDS-PAGE. Immunoblotting was performed with E2F1 and an phospho-PKA substrate antibody.

Since we hypothesized that E2F1 could be a target for PKA, we wanted to show whether these 2 proteins bind to one another. To do this, we performed immunoprecipitation and immunoblotted the membranes with reciprocal antibodies. As shown in Figure 2A, there is a slight association between E2F1 and PKA in the lysate of untreated cells, and the treatment of cells with 20-uM forskolin for 1 h significantly increased the binding of these proteins to each other. After showing the binding, we wanted to show whether PKA would phosphorylate E2F1 under an in vitro condition. To test this, we immunoprecipitated the wild type E2F1 from total cellular lysate prepared from HEK293 cells overexpressing the wild type E2F1. As shown in Figures2B–2C, E2F1 can be effectively immunoprecipitated and phosphorylated by PKA. The phosphorylated E2F1 is identified with anti-PKA substrate antibody, and in vivo phosphorylated E2F1 band in lysate is also evident (Figure 2C). From these results, we concluded that PKA phosphorylates E2F1 under in vitro and in vivo conditions. 

### 3.3. PKA phosphorylation site mutants differentially affect proliferation of HEK293 cells

**Figure 3:**
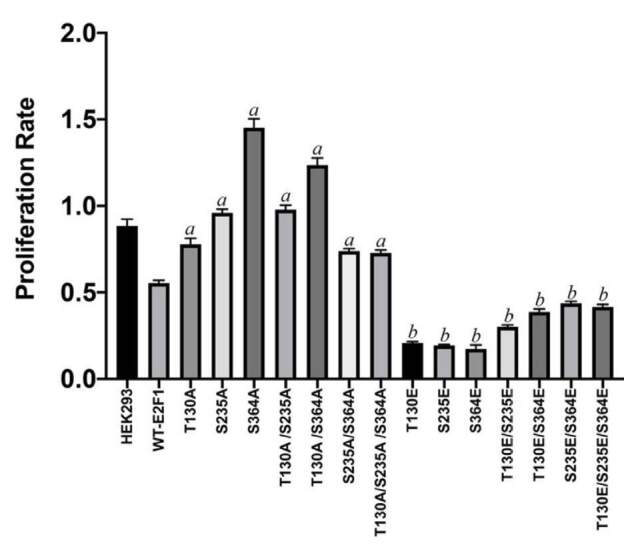
PKA phosphorylation site mutants differentially affect proliferation of HEK293 cells. To determine the effect of Alanine and Glutamic acid mutants of E2F1 on proliferation, HEK293 cells were reverse transfected with 100 ng of mock and vectors in 96-well plates and incubated for 72 h. Cell viability was determined using MTT assay (WT-E2F1 was used as a control in statistical analysis. “a”: statistically significant increase, P < 0.0001; “b”: statistically significant increase, P < 0.0001).

Since the cAMP level goes up in response to deprivation of glucose and nutrients, it is logical to think that activation of PKA under such conditions serves as a defence mechanism. Therefore, under such limiting conditions, the rate of proliferation must be slowed down, and in that respect, the cell cycle must be more tightly regulated or repressed. Therefore, we postulated that Alanine and phosphorylation mimicking glutamic acid mutants of E2F1 would affect the proliferation of cells differently. To test this idea, we transiently transfected the human HEK293 cells with all of the vectors and incubated the cells for 72 h. We then determined cell viability using MTT assay. As shown in Figure 3, ectopic expression of wild type E2F1 diminishes proliferation of HEK293 cells compared to mock transfected cells, and all Alanine mutants, especially S235A, S364A, T130A/S235A, and T130A/S364A mutants, not only prevented the growth-suppressing effect of the wild type E2F1 but also significantly induced the proliferation compared to mock-transfected cells. Moreover, the impact of glutamic acid mutants is even more significant than Alanine mutants, and single glutamic acid mutants diminished proliferation by nearly 80% (Figure 3).

### 3.4. PKA phosphorylation site mutants differentially affect expression of Ki67 in HEK293 cells

**Figure 4:**
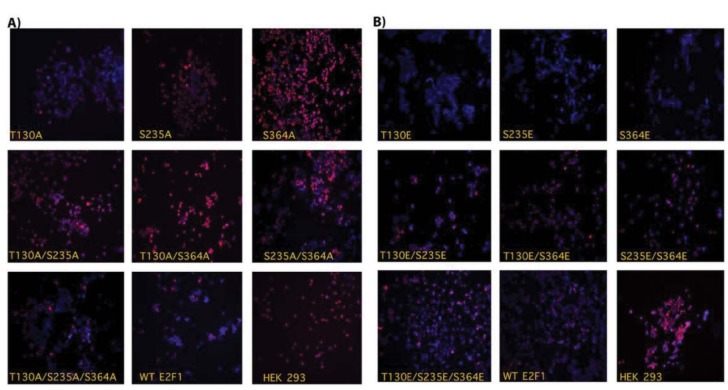
PKA phosphorylation site mutants differentially affect expression of Ki67 in HEK293 cells. HEK293 cells were reverse transfected with 100 ng of mock and vectors in 96-well plates and incubated for 72 h. Then, Ki67 immunofluorescence staining was performed (Ki67: red; DAPI: blue; Merge: red and blue).

After showing the impact of Alanine and Glutamic acid mutants of E2F1 on the proliferation of HEK293 cells, we wanted to compare the effect of all mutants on the expression of proliferation marker Ki67 in HEK293 cells. As shown in Figure 4A, when we looked at the Alanine mutants, we observed perfect alignment between the rate of proliferation with the expression level of Ki67 in HEK293 cells ectopically expressing expression S235A, S364A, S235A/364A, T130A/S235A, and T130A/S364A. However, ectopic expression of wild type E2F1 and T130E, S235E, and S364E mutants significantly diminished the expression of Ki67 compared to mock transfected cells; these results are in complete agreement with the inhibition rate of proliferation of these cells (Figure 4B). Collectively, these results strongly suggest that phosphorylation of E2F1 by PKA at indicated sites dramatically changes the mode of action of E2F1. While PKA-mediated phosphorylation of E2F1 promotes a growth-suppressive effect, Alanine mutants promote cell proliferation.

### 3.5.PKA phosphorylation site mutants differentially affect caspase activation and glucose uptake

**Figure 5:**
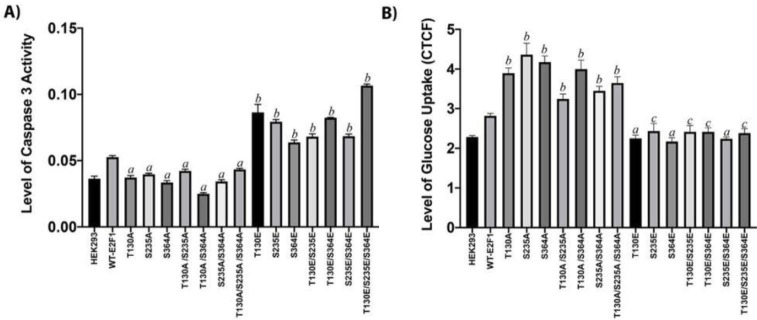
PKA phosphorylation site mutants differentially affect Caspase activation and Glucose uptake. (A) HEK293 cells were reverse transfected with mock-, wild-type, or mutants of E2F1 for 48 h, and caspase-3 activity was determined by colorimetric Caspase-3 activation assay (Bio vision K106-200). (B) HEK293 cells were reverse transfected with mock-, wildtype, or mutants of E2F1 for 24 h in 96-well plates and incubated for 24 h. A Cayman Glucose Uptake Cell Based Assay Kit was used to determine the level of glucose uptake (CTCF: corrected total cell fluorescence). (WT-E2F1 was used as a control in statistical analysis. “a”: statistically significant decrease, P < 0.0001; “b”: statistically significant increase, P < 0.0001; “c”: statistically significant decrease, P < 0.005).

Since we observed that Glutamic acid and Alanine mutants affected the proliferation of HEK293 in almost the opposite way, we wanted to support our observations by determining glucose uptake and activation of caspase-3 in HEK293 cells transiently expressing all mutants. As shown in Figure 5A, ectopic expression of E2F1 slightly increased caspase-3 activity compared to the mock-transfected cell; however, all Glutamic acid mutants, which suppressed the proliferation of HEK293 cells, significantly increased caspase-3 activity, while none of the Alanine mutants induced caspase-3 activity. However, the glucose uptake experiment yielded completely opposite data in relation to caspase-3 activation. While all Alanine mutants significantly induced glucose uptake, all of the Glutamic mutants of E2F1 significantly reduced it, as can be seen in Figure 5B. Taken together, our results strongly suggest that under PKA-activating conditions, E2F1 is phosphorylated by PKA, and this modification suppresses uptake of glucose and pushes the cells toward activation of caspase-3 pathway and suppression of proliferation.

### 3.6. Synchronization of 293T cells using double thymidine block

**Figure 6:**
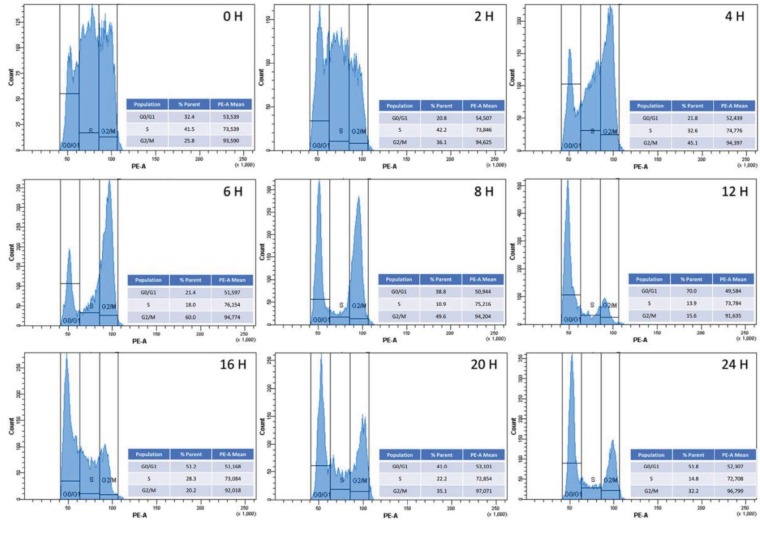
Synchronization of 293T cells using double thymidine block. HEK293 cells were synchronized by double-thymidine block for 48 h; then, the cells were washed with PBS and released by adding complete DMEM (without thymidine). The cells were then collected at 0-2-4-6-8-12-16-20-24 h postrelease for propidium-iodide staining; cell cycle analysis was done using flow cytometry.

E2F1 activity is mainly induced during G1 by CDK-mediated phosphorylation of pRB, and this activation prepares cells to progress from the G1 to S phase. Since E2F1 activity is diminished after the G1 phase, it is important to understand how this is achieved. To shed light on this, we hypothesized that PKA-mediated phosphorylations of E2F1 might play a role in the progression of the cell cycle. To show this, we performed a double thymidine block and arrested the cells at the end of G1, causing a release by resuspending the cells in thymidine-free medium. Cell cycle analysis was done using flow cytometry. As shown in Figure 6, at the time of release (time zero), 32.4% and 41.5% of the cells were arrested at the Go/G1 and S phases, respectively. The G1/S phase-arrested cells progressed very quickly through the late G1 and S phases and at the end of the 4th hour of postrelease, 32% and 45% of the cells were at S and G2/M phases; only 10% of the cells remained at the S phase at the end of the 8th hour of postrelease. At the end of the 12th hour of postrelease, 70%, 13.9%, and 15.6% of the cells were at G0/G1, S, and G2/M phases, respectively. These results indicate that the double-thymidine block protocol arrests these cells very well at the late G1 and early S phases, and progression through the S and G2/M phases can be easily documented.

### 3.7. E2F1 is phosphorylated by PKA at the G0/G1 stages of the cell cycle

**Figure 7:**
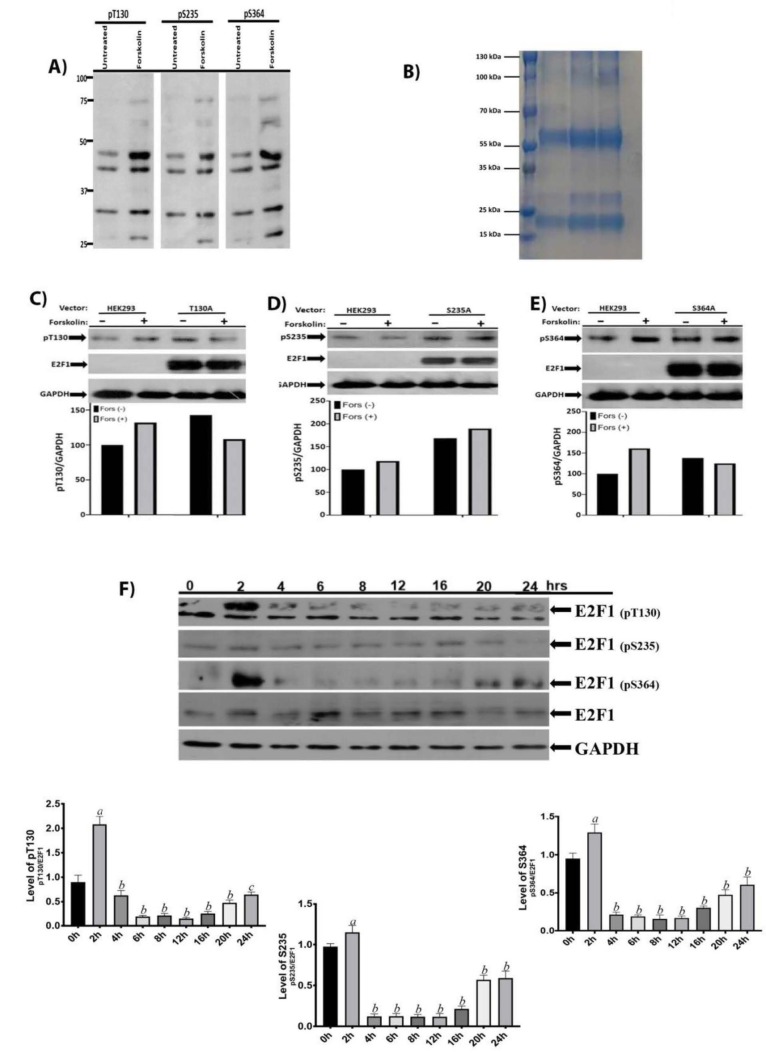
E2F1 is phosphorylated by the PKA at G0/G1 stages of the cell cycle. (A) To determine the specificity of antibodies in our antibodies, we treated serum-starved HEK293 cells with forskolin (20 uM) for 1h, and 100 ug of cell lysates was fractionated on 10%SDS-PAGE; blots were labeled with 1/200 dilutions of the supernatant of our monoclonal hybridomas.(B) Coomasie-blue staining of purified supernatants of our monoclonal hybridomas by column. (C,D,E) We ectopically expressed Alanine mutants of E2F1 for 48h; then, 100 ug of cell lysates were fractionated on 10% SDS-PAGE and blots were labeled with corresponding antibodies. (F) HEK293 cells were synchronized by double-thymidine block for 48 h, released, and the cellular lysates of the released cells were prepared between at 2–24 h. postrelease. 100 mg of total cell lysates were fractionated on 10% SDS-PAGE. Immunoblot was first labeled with anti-E2F1 antibody (1:1000), stripped off, and relabeled with anti-pT130, anti-pS235, and-pS364 antibodies (1:1000) and with antimouse HRP (1:5000). The bottom portion of the blot was also labeled with anti-GAPDH as a loading control. Analysis of SDS-PAGE results: the fold changes in phosphorylation were determined by dividing ImageJ values of band intensities of phospho-bands to that of the E2F1 (zero hour (0 h) protein level was used as a control in statistical analysis. “a”: statistically significant increase, P < 0.0001; “b”: statistically significant decrease, P < 0.0001; “c”: statistically significant decrease, P = 0.0002).

Based on above results, we wanted to determine whether phosphorylation of E2F1 by PKA would align with the progression of the cell cycle through the G0/G1-S-G2/M phases. To test this, we developed monoclonal antibodies against pT130, pS235, and S364 using peptides, (5’-GVKSPGEKSREY- **pT130** -SLNLTTKR-3’:5’-HLMNICTTQLRLL- **pS235** -EDTDSQR-3’:5’-LEQEPLLSRMG- **pS364** -LRAPVDEDR-3’) for immunization. To determine specificity of our antibodies, we prepared lysates of the HEK293 cells untreated or treated with forskolin (20 ug/mL) for 1 h, prepared the lysates and fractionated 100-ug total protein using 10% SDS-PAGE. The blots were labeled with 1/100 dilution of supernatants of monoclonal hybrodomas. As shown in Figure 7A, our antibodies detected the phosphorylated forms of E2F1 at the indicated sites. After that, the supernatants of the monoclonal hybrodomas were collected, and antibodies in the supernatants were purified using an Amicon Pro Affinity Concentration Kit Protein A with 100 kDa colon (ACK5100PA). Commasie-blue staining was performed after SDS-PAGE to examine the protein content of purified antibodies (Figure 7B). To further confirm our results, we ectopically expressed Alanin mutants of E2F1 for 48 h, serum starved the cells for 16 h, treated them with forskolin (20ug/mL) for 1h, and performed western blotting using 100-ug total cell lysates. As shown in Figures 7C–7E, forskolin treatment induced 50% phosphorylations of T130 and S364 by 50%; Alanin mutants diminish these phosphorylations. However, in this experiment we detected very little increase in phosphorylation of S235, and its Alanin mutant did not interfere with this. Also, since we could not detect an endogenous E2F1 level due to the very high level of mutants E2F1, we normalized the phosphoforms to GAPDH to detect induced phosphorylation. 

After confirming the specificity of our antibodies, we used them to detect cell cycle-specific phosphorylations of E2F1. As shown in Figure 7F, T130 and S364 are strongly phosphorylated at the 2nd hour of postrelease, and these phosphorylations progressively declined. Contrary to T130/S364, S235 phosphorylation was minimal at the 2nd hour of postrelease. However, phosphorylations of all 3 amino acids started to increase toward the end of the G2/M phases. Based on these results, we suggest that PKA phosphorylates E2F1 at least at T130 and S364 at the end of the G1 and early S phases. These phosphorylations interfere with the growth-promoting effect ofE2F1 and induces growth-supressing activity of the E2F1 until the cells reach the end of the cell cycle. 

### 3.8. PKA-mediated phosphorylation of E2F1 induces senescence

**Figure 8:**
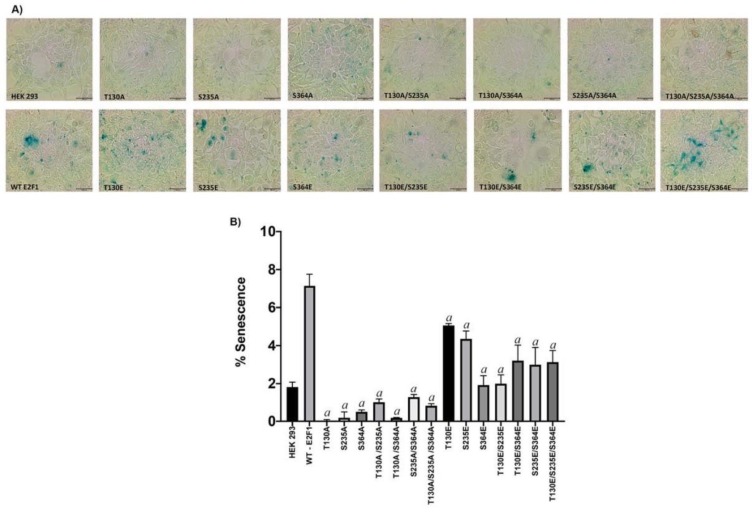
PKA-mediated phosphorylation of E2F1 induces senescence. HEK293 cells were reverse transfected with mock and vectors in 96-well plates and incubated for 48 h. Senescence-associated β-galactosidase staining was then performed. Staining was determined using ImageJ and analyzed with a GraphPad Prism (WT-E2F1 was used as a control in the statistical analysis. “a”: statistically significant increase, P < 0.0001).

Because we observed the opposite effect of Alanine and Glutamic acid mutants of E2F1 on the proliferation of HEK293 cells, we wanted to give further evidence to explain this observation. For this, we tested the effect of the transient expression of the wild type, Alanine, and Glutamic acid mutants of E2F1 on the induction of cellular senescence in HEK293 cells. After 48 h of expression of these proteins, we stained the cells for senescence-associated β-galactosidase activity using X-get al. Since we performed all of the staining on the same plate, we used HEK293 and the wild type E2F1 for both comparisons. As shown in Figures 8A and 8B, ectopic expression of wild type E2F1 induces 3 times more β-galactosidase activity compared to mock-transfected cells; however, all Alanine mutants dramatically inhibited induction of β-galactosidase activity. Particularly, T130A, S235A, and T130A/S364A mutants almost completely ablated the induction of β-galactosidase activity. However, when we looked at Glutamic acid mutants, with the exception of S364E and T130E/S235E mutants, all mutants significantly induced β-galactosidase activity. However, the levels of inductions were less than that of wild type E2F1. These results clearly indicate that the phosphorylation of E2F1 by PKA at indicated sites not only causes the induction of apoptosis but also induces senescence.

## 4. Discussion

The discovery of E2F1 as an E1A-binding protein was a breakthrough inthe regulation of the cell cycle. Until the clarification of posttranslational modifications, pRB remained the main regulator of E2F1 activity. However, in the past 20 years, several posttranslational modifications of E2F1 have been reported and, according to these publications, modifications affect either the function or the stability of E2F1 (Ertosun et al., 2016). In addition to these studies, Bagchi et al. reported that cAMP-dependent kinase (PKA)-mediated phosphorylation of E2F1 induced its DNA-binding capacity. Although this has been known since 1989, PKA-mediated phosphorylation sites and the significance of these phosphorylations on cellular physiology have not been fully elucidated. 

The cyclic AMP-dependent protein kinase (PKA) plays a significant role in cellular physiology. Activation of PKA is induced by cAMP, which is synthesized by adenylate cyclase when the cellular glucose level drops. Therefore, PKA is considered to be a glucose sensor. Activated PKA elicits its activity on many cellular events such as the uptake and metabolism of glucose (Jensen, 2007; Kaihara et al., 2015), cell proliferation and progression of the cell cycle throughG1 to S, and completion of the M phase (Anghileri et al., 1999; Costanzo et al., 1999). Although the involvement of PKA in these cellular events has been documented, the molecular mechanism or molecular target(s) behind these regulations have not yet been addressed. 

With this in mind and knowing that E2F1 is also involved in the regulation of these cellular processes, we wanted to determine whether the modifications of E2F1 by PKA would have an effect on cellular events such as the proliferation, glucose uptake, and progression of the cell cycle. As mentioned before, we found 3 putative PKA-phosphorylation sites located at T130, S235, and S364, and converted the codons of these amino acids to nonphosphorylatable Alanine and phosphorylation mimicking Glutamic acid on eukaryotic the E2F1 expression vector we created. Since the stability of E2F1 seems to be affected by the previously mentioned modifications, we first wanted to determine whether adenylate cyclase activator forskolin-mediated activation of PKA would have an effect on the stability of E2F1. Indeed, as shown in Figure 1A, stimulation of HEK293 cells with 20 mM forskolin affected stability and caused a 75% drop in the E2F1 level at the 16th hour of poststimulation. However, after 16 h, the E2F1 level gradually increased and reached a basal level. To support the involvement of PKA in the destabilization of E2F1, we pretreated HEK293 cells with PKA inhibitor H9 for 1 h prior to forskolin treatment.This pretreatment significantly inhibited the forskolin-mediated destabilization/degradation of E2F1, as seen in Figure 1B. These results clearly indicate that the activation of PKA is responsible for the destabilization of E2F1. 

After determining the effect of PKA activation on the stability of E2F1, we next showed the expression levels of all of the mutants of E2F1 in transiently transfected HEK293 cells. As shown in Figures 1C and 1D, all mutants of E2F1 were expressed at very high levels in the HEK293 cells for at least 48 h. The expression levels of E2F1 mutants were very high compared to the basal level of expression. Therefore,we did not omit the basal level of expression of E2F1 for other experiments. After showing the ectopic expressions of all mutants, we wanted to determine whether PKA and E2F1 would associate with one another under normal growth conditions. To avoid forced associations, we immunoprecipitated both proteins from untransfected HEK293 cells, and immunoblots were labeled with reciprocal antibodies. As anticipated, we found strong in vivo associations of E2F1 and PKA even in the absence of forskolin, indicating that E2F1 serves as substrate of PKA under normal growth conditions. After demonstrating the physical association, we used the same samples to show direct phosphorylation of E2F1 by PKA and found that PKA effectively phosphorylates E2F1 under in vitro conditions, as seen in Figure 2C. Collectively, these results clearly show that PKA binds to and phosphorylates E2F1. 

As we mention above, the main aim of our study was to show that the antiproliferative activity of PKA could be mediated, at least in part, by PKA-mediated E2F1 phosphorylation. Therefore, we focused on the impact of PKA phosphorylation site mutants of E2F1 on cell proliferation, apoptosis, senescence, and glucose uptake. As anticipated, ectopic expression of all Alanine mutants induced the proliferation of HEK293 and induced the expression of the proliferation marker of Ki67, as well as glucose uptake. The same mutants suppressed Caspase-3 activation and senescence. Contrary to this, Glutamic acid mutants produced almost completely opposite results.Therefore, we suggest that PKA-mediated phosphorylation of E2F1 induces a growth suppressive effect. Although Alanine mutants are artificial proteins, the results produced from these mutants strongly support our hypothesis. 

E2F1 is sequestered in its inactive form during G0/G1 by forming a complex with pRB and are released/activated after CDK-mediated phosphorylation of pRB at the G1 phase. The activated E2F1 prepares cells to enter the S phase by inducing the expression of many genes responsible for DNA synthesis and progression of cell the cycle through the S-G2/M phases (Ertosun et al., 2016). Since chemical modifications can affect the function of E2F1 and because our Glutamic acid mutants inhibited cell proliferation and induced senescence, we wanted to determine the exact time point at which E2F1 phosphorylation takes place during the cell cycle. To address this, we first developed monoclonal antibodies against phosphopeptides of E2F1 bearing pT130, pS235, and pS364. Next, we synchronized HEK293 cells by using a double-thymidine block, which arrests cells at the end of the G1 and early S phases (Chen and Deng, 2018).We determined the phases of the cell cycle by using propidium iodide staining. After determining the phases of the cell cycle, we prepared the protein lysates of cells synchronized the same way and for the same time points and labeled the immunoblots using our monoclonal antibodies. As shown in Figure 7F, T130 and S364 were heavily phosphorylated immediately after the release of cells from the thymidine block. These phosphorylations gradually declined. These results indicate that PKA targets E2F1 at the late G1 and early S phases. However, these phosphorylations were kept at a minimal level during the S and G2/M phases. Contrary to T130 and S364 phosphorylations, PKA does not seem to phosphorylate S235 heavily, although the phosphorylation pattern of S235 is very similar to T130 and S364. Since PKA-induced phophorylations seem to suppress the proliferation inducing activity of E2F1, our results are in good agreement with the required activity of E2F1 during the S phase.

Collectively, our results suggest that E2F1 serves as a substrate of PKA under in vivo conditions. In addition, PKA-mediated phosphorylations of E2F1 may be used to silence the growth-promoting activity of E2F1 after the completion of the G1 and S phases. In this way, the cell is prepared to enter G0 for initiation of another cell cycle. Our results significantly contribute to our understanding of the molecular mode of action of E2F1 as the master regulator of the cell cycle. This explains how PKA-mediated phosphorylation of E2F1 causes the suppression of cell proliferation, suppression of glucose uptake, and induction of caspase-3 activation and senescence in HEK293 cells. 

Supplementary Materials
