# Supplementary figures and images for: Regulation of E2F1 activity via PKA-mediated phosphorylations
Source: Turk J Biol. 2020 Oct 13;44(5):215–29. doi: 10.3906/biy-2003-9 (PMC7585165; doi:10.3906/biy-2003-9)

Supplemental Figures

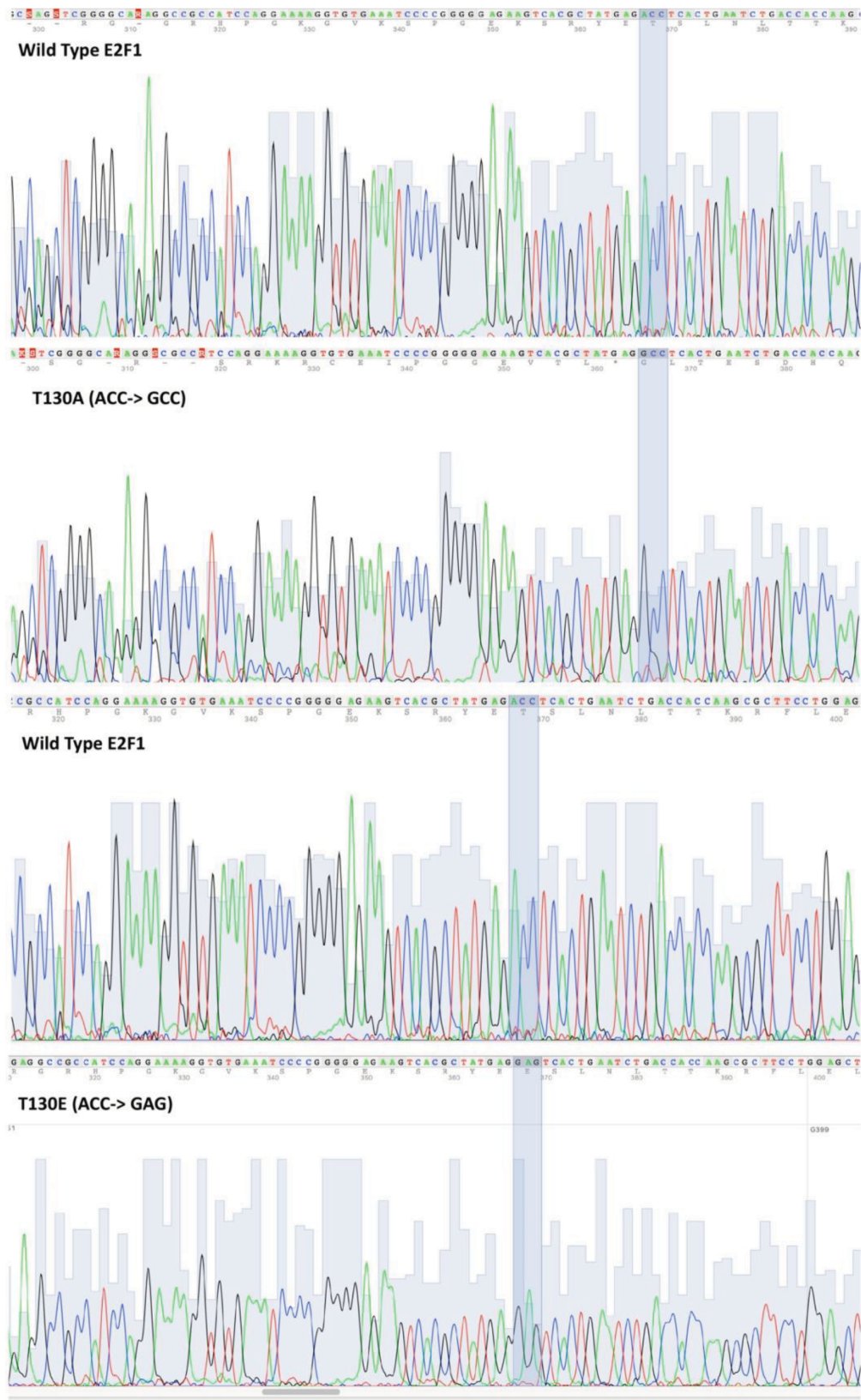

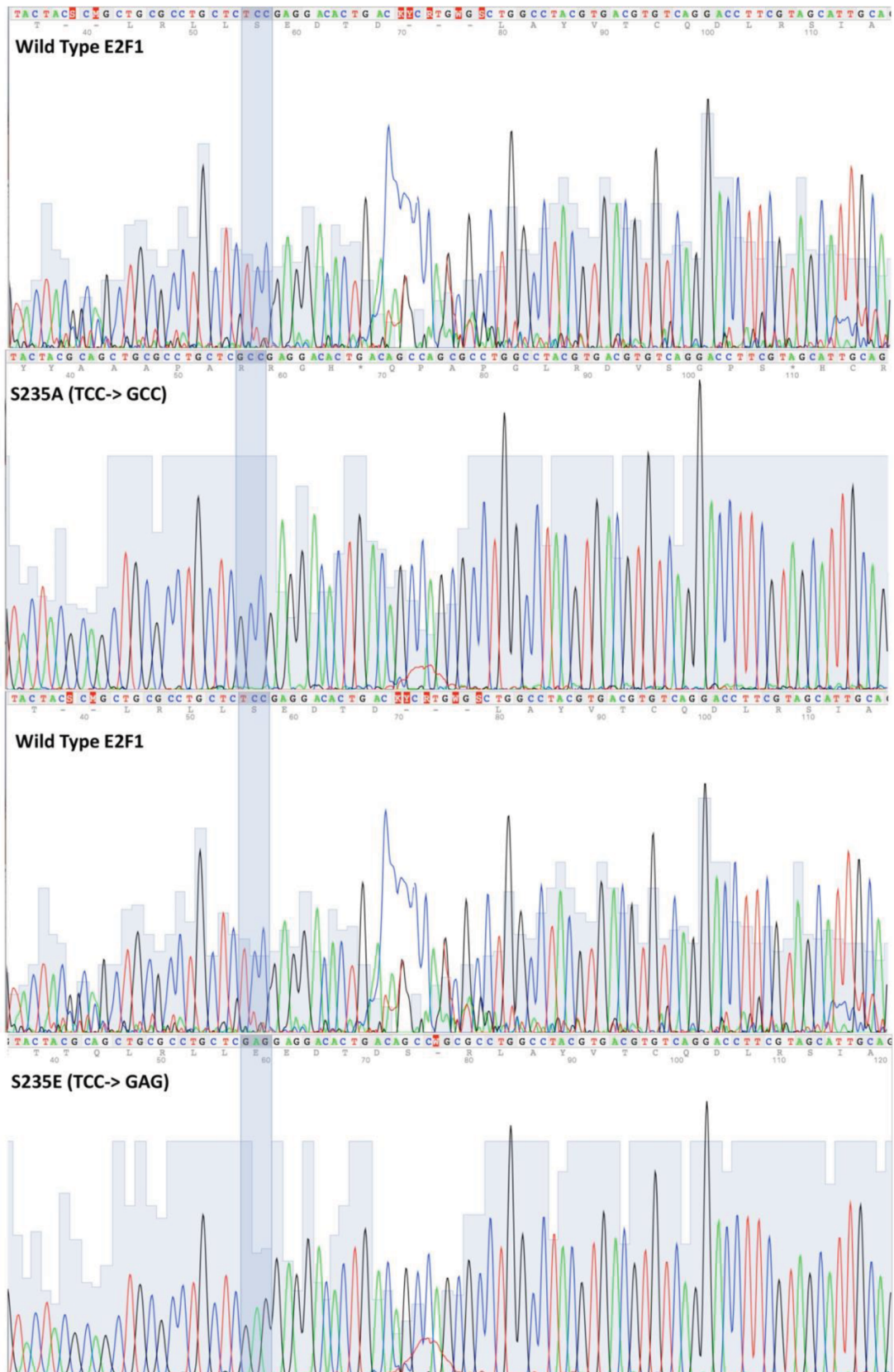

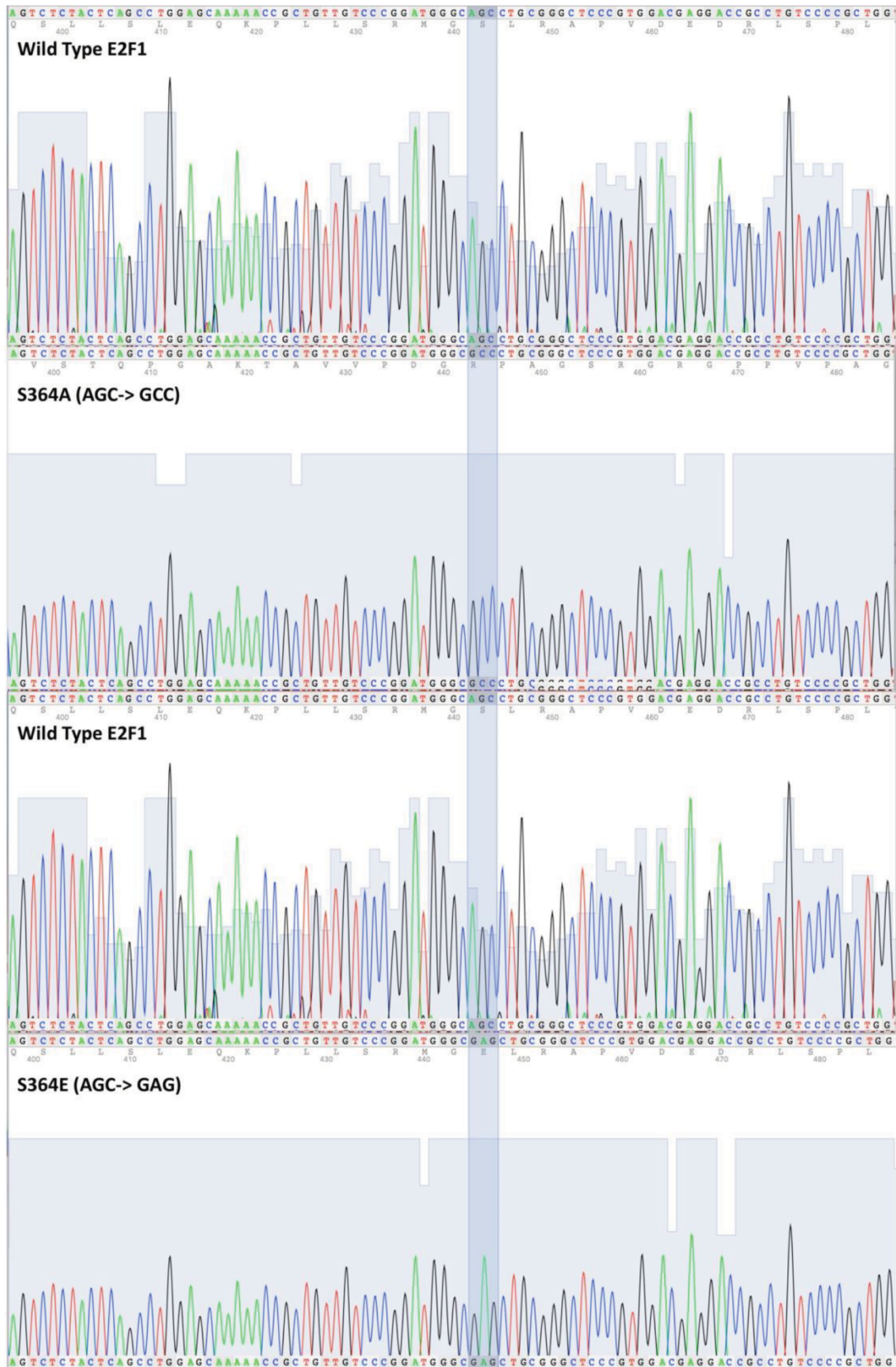

Supplement: Supplementary file 1 — Supplementary Materials [file turkjbio-44-215-sup001.pdf]
